# Long-term outcomes of two types of metal stent for chronic benign ureteral strictures

**DOI:** 10.1186/s12894-019-0465-5

**Published:** 2019-05-06

**Authors:** Joongwon Choi, Kyung Jin Chung, Seol Ho Choo, Deok Hyun Han

**Affiliations:** 1 Department of Urology, Samsung Medical Center, Sungkyunkwan University School of Medicine, 81 Irwon-ro, Gangnam-gu, Seoul, 06351 South Korea; 2 Department of Urology, Gachon University Gil Medical Center, Gachon University School of Medicine, 21, Namdong-daero 774beon-gil, Namdong-gu, Incheon, 21565 South Korea; 3 Department of Urology, Ajou University Medical Center, Ajou University School of Medicine, 164, World cup-ro, Yeongtong-gu, Suwon, 16499 South Korea

**Keywords:** Benign ureteral stricture, Mesh, Metal stent, Success rate, Thermo-expandable

## Abstract

**Background:**

We aimed to compare the results of long-term use of two types of metal stent for chronic benign ureteral strictures.

**Methods:**

Our study included 46 ureter units (UUs) that underwent metal stent placement from 2010 to 2017. We included benign ureteral strictures causes by variety reasons that could not be solved by other treatment and malignant obstructions were excluded. Covered mesh stent (Uventa™) and a thermo-expandable stent (Memokath 051™) were used. Primary success was defined as maintaining patency without procedures and overall success was defined as maintaining patency with additional procedures.

**Results:**

We placed covered mesh stents in 25 UUs and thermo-expandable stents in 21 UUs. The mean follow-up duration of each stent was 41.4 ± 23.1 and 34.4 ± 16.5 months (*p* = 0.250). In the first year of stent insertion, primary success was achieved in 54.9 and 70.4% (*p* = 0.204). Overall success was achieved in 78.7 and 75.4% in same duration, respectively (*p* = 0.586). Longer stent placement had positive predictive value on both success rates (HR = 0.185, *p* = 0.047 and HR = 0.111, *p* = 0.018). Prior radiation therapy and non-pelvic ureter stricture both adversely affected the overall success rate (HR = 5.412, *p* = 0.048 and HR = 4.203, *p* = 0.030). Previous PCN status had negative predictive value for both success rates (HR = 4.014, *p* = 0.003 and HR = 3.064, *p* = 0.035).

**Conclusions:**

The treatment outcomes of two types of metal stent were comparable, especially in the first year of stent insertion.

**Electronic supplementary material:**

The online version of this article (10.1186/s12894-019-0465-5) contains supplementary material, which is available to authorized users.

## Background

Ureteral strictures can occur in a wide variety of benign and malignant diseases. Benign strictures have various etiologies, including radiation therapy, retroperitoneal fibrosis, and ureter stones. When urinary diversion is not effective, ureteral strictures can turn chronic, causing serious problems. Conventional polymer stents can also introduce several problems, such as encrustation, stone formation, pain, infection, reflux and migration [1]. Open urinary diversions were gradually abandoned because of their technical difficulty with associated risk [2]. After metal stents were successfully used in vascular and biliary systems, they began to be used in urinary diseases. Milroy et al. [3] inserted the first metal stents in urethral stricture patients in 1988. Since then, many metal stents have been developed, but identifying the best stent for specific conditions is difficult because each stent has a different mechanism.

Malignant ureteral strictures are caused by extrinsic compression or direct invasion by a primary or metastatic tumor and lymphadenopathy [4]. The nature of strictures caused by direct tumor invasion can be very different from strictures caused by benign diseases.

Comparing the effects of metal stents in a benign environment allows us to eliminate complexity and make relatively objective comparisons. Thus, we compared two metal stent types—the covered mesh stent and the thermo-expandable stent. The Uventa™ model (Taewoong Medical, Seoul, Korea) was chosen as the mesh stent, and the Memokath 051™ model (PNN Medical, Glostrup, Denmark) was chosen as the thermo-expandable stent.

Early metal stent models had a problem with tissue ingrowth, which led to ureteral lumen occlusion and increased the difficulty of endoscopic removal [5]. To solve this problem, an externally coated single-layered segmental stent (Passager; Boston Scientific, Miami, FL, USA) was developed for ureteral strictures. However, the polytetrafluoroethylene (PTFE) membrane covering the stent reduced the stent’s ability to obtain firm adhesion, which caused high rates of stent migration—as much as 81.0% [6].

Uventa was developed in response to these problems. It has a triple-layered mesh construction, and the PTFE is stacked with nitinol above and below. The outer stent has a high friction coefficient to prevent migration, while the PTFE membrane prevents tissue ingrowth [7]. Its diameter is 7 mm, and the length ranges from 6 to 16 cm.

Memokath 051™ has a closed spiral structure which can prevent urothelial ingrowth. Its tight spiral and metal alloy hold the stricture site without urothelial overgrowth [5]. Its titanium component has the ability to resist corrosion in the urinary system [1]. Stoller et al. [8] reported that the spiral stent yielded higher flow rates than the conventional polymer stent. The products are available in lengths of 3, 6, 10, 15, 20, and 25 cm.

A previous study compared the two stents [9], but did not distinguish between benign and malignant causes, and complications were not systematically classified. The study also did not compare the primary success rate with the overall success rate. The aim of this study is to improve those limitations and compare two type of stent, and identified the risk factors for keep patency.

## Methods

The Institutional Review Board of Samsung Medical Center approved this study (IRB No.: 2018–05–106-001). We retrospectively obtained data from the medical records of patients who presented with chronic benign ureteral strictures and received metal stent placement. From May 2010 to December 2017, 36 patients (14 men and 22 women), with a total of 46 ureter units, underwent metal stent placement. We included benign ureteral strictures that were caused by radiation therapy, retroperitoneal fibrosis, ureter stone, pelvic surgery, ureteropelvic junction (UPJ) obstruction, renal tuberculosis, fibromatosis, and pelvic abscess. We excluded obstructions caused by direct malignant invasion.

To compare the efficacy and safety of mesh and thermo-expandable stents, we examined all medical records, including age, gender, previous pelvic radiation therapy (RT), side (right or left ureter), stricture length, lesion crossing the ureterovesical (UVJ) junction, previous balloon dilatation, previous double J (D-J) stenting, and previous percutaneous nephrostomy (PCN) catheter insertion.

We tended to insert a mesh stent if the sticture length was long (> 10 cm) or expected to be difficult to insert, which is easier to insert because it allows us to insert separate smaller stents.

We defined primary success as maintaining patency after the first stenting without an additional procedure. And overall success was defined as maintaining patency after further salvage procedures during the observed period. For example, if patency was maintained after the stent change or removed small debris from the ureteroscopy, it was considered as an overall success. Chung et al. first proposed this methodology [10].

Baumgarten et al. [11] defined stent failure as a need for PCN insertion, increasing hydronephrosis with a metal ureteral stent, or deteriorating renal function that is suspected to be post-renal in nature. We define this as failure. If patient have to keep a PCN with a metal stent, it is considered a failure.

We divided the ureteral stricture location into a pelvic ureter and non-pelvic ureter, according to the international anatomical system.

### Statistical analysis

All results are presented as number with percent, mean with standard deviation, or median with interquartile range. We used the Kolmogorov-Smirnov statistic to analyze continuous variables for normality. We used the Mann-Whitney U-test to analyze descriptive variables, except age, stricture length, and follow-up months, which were calculated using the independent t-test. We estimated time to primary and overall failure using Kaplan-Meier curves and the log-rank test. We analyzed risk factors for primary and overall success with Cox regression analysis. Data were analyzed using SPSS version 21.0 (IBM, Chicago, IL, USA) and MedCalc version 14 (MedCalc Software, Ostend, Belgium).

## Results

Table 1 shows the basic clinical characteristics. There were no differences between the groups except prior radiation therapy (RT, *p* = 0.008) and stricture length (*p* = 0.010). The most common motivation for replacement of the existing stent with a metal stent was inconvenience of frequent replacement (56.0 and 76.2%) followed by D-J malfunction (36.0 and 19.0%). Few people wanted to change to a metal stent to manage irritation symptoms from their existing D-J stent.

**Table 1:** Basic clinical characteristics of the mesh and thermo-expandable stent groups

*Characteristics*	*Mesh (n = 25)*	*Thermo-expandable (n = 21)*	*p-value*
Age, yr	62.7 ± 15.3	59.1 ± 12.6	0.394
Gender, n (%)			0.430
Male	9 (36.0)	10 (47.6)	
Female	16 (64.0)	11 (52.4)	
Previous pelvic radiation therapy	13 (52.0)	3 (14.3)	0.008
Side, n (%)			0.980
Right	13 (52.0)	11 (52.4)	
Left	12 (48.0)	10 (47.6)	
Stricture length (cm)	12.7 ± 6.3	7.8 ± 6.0	0.010
Reason for placement			0.159
D-J malfunction	9 (36.0)	4 (19.0)	
Irritation symptoms of D-J	2 (8.0)	1 (4.8)	
Inconvenience of frequent replacement	14 (56.0)	16 (76.2)	
Prior diversion, n (%)			
D-J	24 (96.0)	19 (90.5)	0.455
PCN	12 (48.0)	13 (61.9)	0.351

*D-J* double-J stent, *PCN* percutaneous nephrostomy

The underlying causes of metal stent placements are presented in Table 2. Radiation therapy was the most common cause (40.0%) for mesh stenting, followed by idiopathic (20.0%) and retroperitoneal fibrosis (20.0%). For thermo-expandable stenting, pelvic surgery was the most common cause (28.6%).

**Table 2:** Underlying Causes of Chronic Benign Strictures

	*Mesh (n = 25)*	*Thermo-expandable (n = 21)*
Idiopathic	5 (20.0)	1 (4.8)
Radiation therapy	10 (40.0)	3 (14.3)
Retroperitoneal fibrosis	5 (20.0)	4 (19.0)
Ureter stone	0 (0.0)	4 (19.0)
Pelvic surgery	2 (8.0)	6 (28.6)
UPJ obstruction	0 (0.0)	2 (9.5)
Renal tuberculosis	0 (0.0)	1 (4.8)
Fibromatosis	1 (4.0)	0 (0.0)
Pelvic abscess	2 (8.0)	0 (0.0)

*UPJ* ureteropelvic junction

Intraoperative factors are compared in Table 3. The two stent groups were statistically different with regard to stent length (*p* = 0.008). Mesh stents are technically capable of being inserted as a set of overlapping multiple stents, while thermo-expandable stents are designed to be inserted as a single.

**Table 3:** Intraoperative Factors of the Mesh and Thermo-Expandable Stent Groups

*Characteristics*	*Mesh (n = 25)*	*Thermo-expandable (n = 21)*	p*-value*
Stent length			0.008
< 10 cm	2 (8.0)	9 (42.9)	
10–15 cm	12 (48.0)	8 (38.1)	
> 15 cm or multiple^a^	11 (44.0)	4 (19.0)	
Location			0.060
Upper	2 (8.0)	4 (19.0)	
Mid	1 (4.0)	4 (19.0)	
Lower	4 (16.0)	5 (23.8)	
Upper-mid	5 (20.0)	1 (4.8)	
Mid-lower	4 (16.0)	2 (9.5)	
Upper-lower	9 (36.0)	5 (23.8)	
Across UVJ	10 (40.0)	4 (19.0)	0.128
Ballooning			0.521
Before stent placement	4 (16.0)	7 (33.3)	
After stent placement	5 (20.0)	0 (0.0)	

^a^more than two stents

*UVJ* Ureterovesical junction

**Fig. 1:**
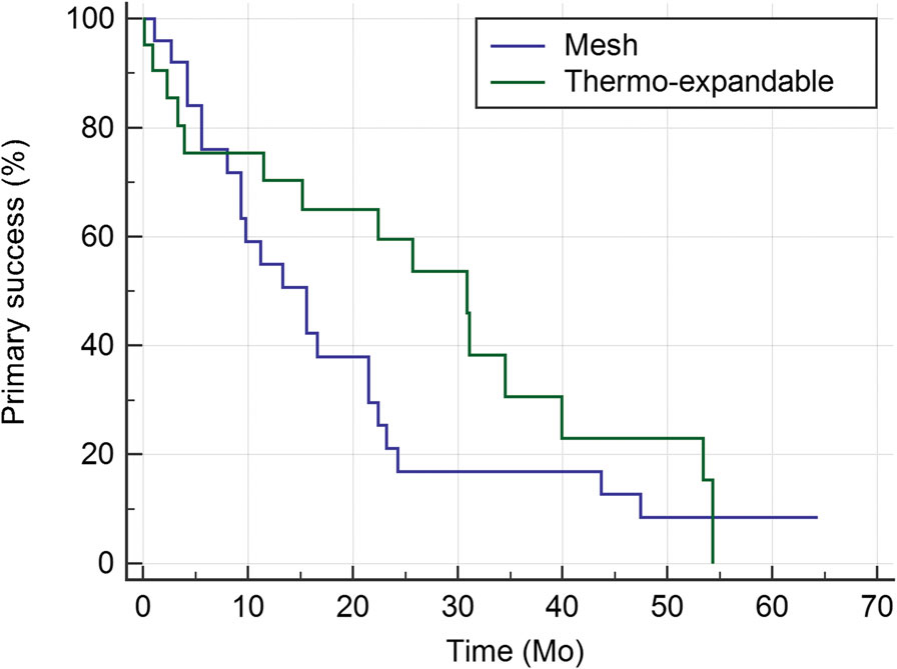
Kaplan-Meier curves for primary success rates of the mesh and thermo-expandable metal stents: The medians (95% CI) for the two groups were 15.6 (9.3–21.5) and 30.9 (15.2–39.9), respectively (*p* = 0.204)

**Fig. 2:**
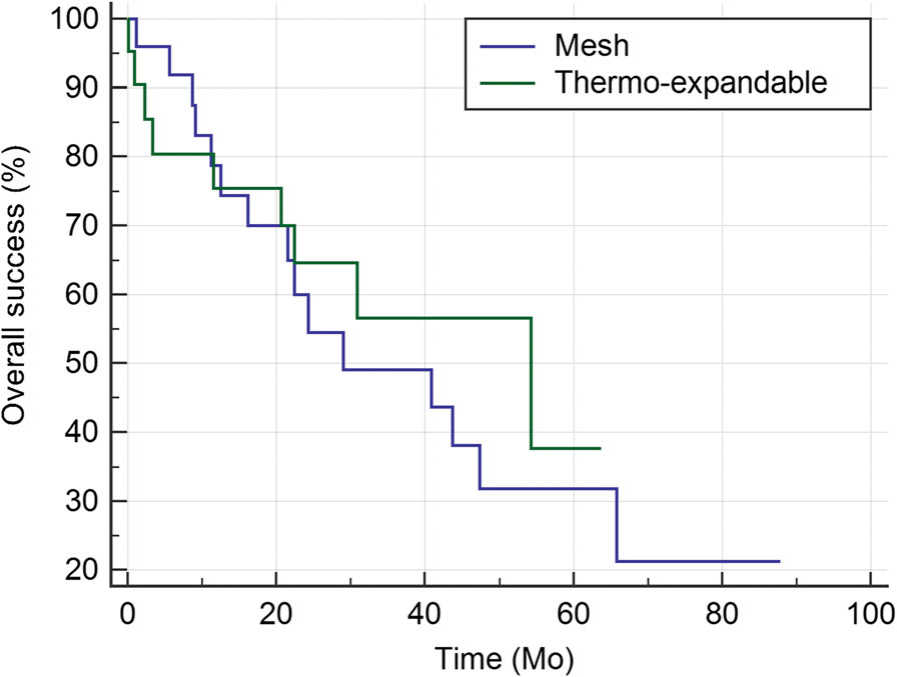
Kaplan-Meier curves for overall success rates of the mesh and thermo-expandable metal stents: The medians (95% CI) for the two groups were 29.0 (21.5–65.8) and 54.3 (20.6–54.3), respectively (*p* = 0.586)

Time to primary failure is presented in Fig. 1. The median time to primary failure was 15.6 (9.3–21.5) months for mesh stents and 30.9 (15.2–39.9) months for thermo-expandable stents (*p* = 0.204). Figure 2 shows time to overall failure. The median time to overall failure was 29.0 (21.5–65.8) and 54.3 (20.6–54.3) months, respectively (*p* = 0.586).

The treatment outcomes for the mesh and thermo-expandable stent groups were comparable (Table 4). There was no statistical difference in follow-up months between the two groups (*p* = 0.250). Primary success was achieved for 12.0% of mesh stents and 28.6% of thermo-expandable stents throughout the entire observation period (*p* = 0.204). The overall success rates were 40.0 and 57.1%, respectively (*p* = 0.586). Thermo-expandable stents had better durability for primary success, especially in the second and third years.

**Table 4:** Treatment Outcomes of the Mesh and Thermo-Expandable Stent Groups

	*Mesh (n = 25)*		*Thermo-expandable (n = 21)*	
	*Primary*	*Overall*	*Primary*	*Overall*
Follow-up months^a^	41.4 ± 23.1		34.4 ± 16.5	
Success rate^b^, n (%)				
First year (%)	13 (54.9)	18 (78.7)	14 (70.4)	15 (75.4)
Second year (%)	4 (16.9)	10 (54.5)	11 (59.5)	12 (64.6)
Third year (%)	4 (16.9)	9 (49.1)	4 (30.6)	7 (56.5)
Time to 50% failure (month)	15.6	29.0	30.9	54.3
Cause of failure, n (%)				
Patency-related failure				
Migration	4 (16.0)	4 (16.0)	2 (9.5)	1 (4.8)
Hyperplasia	14 (56.0)	5 (20.0)	3 (14.3)	5 (23.8)
Encrustation	1 (4.0)	2 (8.0)	9 (42.9)	3 (14.3)
Patency-unrelated failure				
Concomitant change^c^	0 (0.0)	0 (0.0)	1 (4.8)	0 (0.0)
Fistula	2 (8.0)	3 (12.0)	0 (0.0)	0 (0.0)
Pseudoaneurysm	1 (4.0)	1 (4.0)	0 (0.0)	0 (0.0)

^a^Follow-up months for the two groups were comparable (*p* = 0.250)

^b^Success rate is an estimate, and the standard error is omitted. Neither the primary nor the overall rates were statistically significant (*p* = 0.204, 0.586)

^c^Concomitant change refers to the exchange of one obstructed stent as well as the other unobstructed stent due to concerns over future obstruction

Table 5 shows the risk factors affecting primary and overall success. Although stent type did not have a statistically significant effect on success rate, thermo-expandable stents generally yielded more favorable results for primary success (HR = 0.393, *p* = 0.052). Previous PCN status negatively impacted both primary (HR = 4.014, *p* = 0.003) and overall success (HR = 3.064, *p* = 0.035). However, longer stents had a positive effect on primary (HR = 0.185, *p* = 0.047) and overall success (HR = 0.111, *p* = 0.018). Prior radiation therapy and non-pelvic ureteral stricture adversely affected the overall success rate (HR = 5.412, *p* = 0.048). Additionally, risk factors of mesh and thermo-expandable stent were analyzed separately. The female gender was identified as the protective factor of primary success (HR = 0.136, *p* = 0.024) in mesh stent (Additional file 1: Table S1). In thermo-expandable stent, non-pelvic ureter (HR = 6.134, p = 0.048), previous PCN (HR = 10.192, *p* = 0.013) was risk factors (Additional file 2: Table S2) and stent length more than 15 cm reduce risk of primary failure (HR = 0.010, *p* = 0.037). Factors that showed multicollinearity were excluded.

**Table 5:** Risk Factors Affecting Primary and Overall Success Rates

	*Primary*		*Overall*	
	*HR*	p*-value*	*HR*	p*-value*
Stent type				
Mesh	1.000	─	1.000	─
Thermo-expandable	0.393	0.052	0.680	0.468
Age (yr)	0.983	0.298	0.966	0.095
Gender				
Male	1.000	─	1.000	─
Female	0.469	0.131	0.640	0.462
Stricture location				
Pelvic ureter	1.000	─	1.000	─
Non-pelvic ureter^a^	2.631	0.062	4.203	0.030^*^
Stricture length (cm)				
≤10 cm	1.000	─	1.000	─
> 10 cm	2.925	0.077	1.789	0.430
Stent length		0.138		0.058
< 10 cm	1.000	─	1.000	─
10–15 cm	0.425	0.144	0.388	0.140
> 15 cm or multiple^b^	0.185	0.047^*^	0.111	0.018^*^
Prior radiation therapy				
No	1.000	─	1.000	─
Yes	2.155	0.198	5.412	0.048^*^
Previous PCN				
No	1.000	─	1.000	─
Yes	4.014	0.003^*^	3.064	0.035^*^
Balloon dilatation				
No	1.000	─	1.000	─
Yes	0.980	0.963	0.978	0.970

^*^Statistically significant (*p* < 0.05)

^a^upper ureteral stricture only

^b^more than two stents

*HR* hazard ratio, *PCN* percutaneous nephrostomy

Table 6 shows the types and numbers of failure events for both stents. If migration occurred but patency was maintained, we did not define it as a failure; this was relevant for one case in our study. The number of complication events was 31 for mesh stents and 15 for thermo-expandable stents; this difference was borderline significant (*p* = 0.08). The numbers of severe complications (above grade 3) were 12 and 10, respectively, which were not significantly different (*p* = 0.96).

**Table 6:** Numbers of All Types of Complication Events

*Complications*	*Modified Clavien classification*						
	*Total*	I	II	IIIa	IIIb	IV	V
Mesh (*n* = 25)		8	11	1	9	1	1
Persistent pain	3 (12.0%)	3					
Lower urinary tract symptoms	5 (20.0%)	5					
Urinary tract infection	9 (36.0%)		8				1
Persistent hematuria	3 (12.0%)		3				
Ureteroenteric fistula	3 (12.0%)				2	1	
Stent migration	5 (20.0%)				5		
Encrustation	2 (8.0%)				2		
Iliac artery pseudoaneurysm	1 (4.0%)			1			
Thermo-expandable (*n* = 21)		2	3	0	10	0	0
Persistent pain	1 (4.8%)	1					
Lower urinary tract symptoms	1 (4.8%)	1					
Urinary tract infection	2 (9.5%)		2				
Persistent hematuria	1 (4.8%)		1				
Ureteroenteric fistula	0 (0.0%)						
Stent migration	3 (14.3%)				3		
Encrustation	7 (33.3%)				7		
Iliac artery pseudoaneurysm	0 (0.0%)						

## Discussion

There have been a number of conflicting evaluations of metal stents. While early studies were generally promising [12–14], the reports shifted from mixed to unfavorable for both mesh [7, 9, 10] and thermo-expandable stents [2].

We used metal stents for benign strictures because we experienced acceptable results with malignant obstructions. In Korea, benign ureteral stricture is included in the indication of metal stent placement and It has also been reported that the metal stent is effective in the benign ureteral stricture [15]. Patients included in this study had sufficient discussion on the choice of treatment policies, and include cases who do not want to have repeated reconstructive surgery or it was not adaptive. Two UPJ stricture included in this study were recurred after pyeloplasty cases. One case was recurred after an additional endoureterotomy, and the other was who had history of multiple balloon dilatation after recur. The treatment policy was determined after sufficient consultation.

Since urinary tract reconstruction is not possible for every patient, metal stenting became a useful option in our practice. We wanted to identify effective metal stent for chronic benign ureteral strictures, especially between two commonly used stent in our hospital. There were no significant differences in primary (*p* = 0.204) and overall (*p* = 0.586) success rates between the mesh and thermo-expandable stents, and both stent types are suitable for treating chronic benign ureteral strictures.

Although not statistically significant, the thermo-expandable stent yielded a better primary success rate in the first, second, and third years, and the time to 50% failure was generally longer for thermo-expandable stents. However, there were no major differences in the overall success rates.

Previous PCN was a factor against both primary (HR = 4.014, *p* = 0.003) and overall (HR = 3.064, *p* = 0.035) success. This was not a direct problem with PCN itself; rather most patients with PCN had a severe stricture which was impossible to place a D-J stent under local anesthesia.

It is interesting that stent length longer than 15 cm and multiple stent insertions were positive factors for primary and overall success (HR = 0.185, *p* = 0.047 and HR = 0.111, *p* = 0.018). We suspect that sufficient lengthy stent insertion warrants favorable results rather than inserting a short stent that fits in a stricture segment.

Our study shows that the success rate is high when including the pelvic ureter. According to our search, the risk difference in stricture location is not clear. Pelvic ureter receives blood supplies from a much wider variety of vessels than abdominal ureter such as internal iliac artery, superior vesical artery, middle rectal artery, and inferior vesical artery. Which may have an effect, but further research is needed.

Consistent with previous studies, we found that prior radiation therapy decreased the likelihood of overall success [HR = 5.412, *p* = 0.048] [4, 7]. However, Liatsikos et al. [16] evaluated the effect of external RT in a pig model. They found that RT was safe for tissue containing a metal stent, but placing a metal stent in an ureter which was weakened by previous RT may cause tissue damage.

In our study, age was not a risk factor for metal stent failure. However, another study discussed age-related biomechanical changes in the ureter. According to Petsepe et al. [17], the muscle layer thickens with age, unlike the ureteral epithelium and lamina propria. Also, a histological animal study of metal stents found that the stent wire severely compressing ureteral epithelium [18]. It is possible that resistance of the ureter to a metal stent could decrease with age.

The overall trend was similar when risk factors were verified according to the stent type, but there were some differences in two groups. Mesh stent showed favorable results when used in female gender (HR = 0.136), and was not affected by other risk factors. On the other hand, the primary failure was increased in the non-pelvic ureter with thermo-expandable stent (HR = 6.134). And it showed a favorable result when stent longer than 15 cm was inserted (HR = 0.010). In subgroup analysis, there were no factors affecting overall success.

Hsu et al. [19] reported antegrade insertion and response to cancer treatment as factors predicting immediate stent failure. Kim et al. [7] reported that female, cervical cancer, stricture length over 6 cm, and post-procedure follow-up over two years were risk factors for severe complications.

The mesh stent tended to caused more severe complications in our study. Overall, three fistulas and one pseudoaneurysm occurred in the mesh stent group. One patient had a stent placement because of radiation fibrosis, and the stent was exposed to the rectum for 21.5 months after the procedure, which was removed by open surgery. Another patient also had radiation fibrosis, and a left ureterosigmoid fistula was discovered only 9.3 months after the procedure; emergency exploration was performed and stent was removed. Another patient visited the emergency room with gross hematuria 13.3 months after metal stent insertion. In this case, CT findings revealed a pseudoaneurysm along the left distal ureter to the proximal sigmoid colon; the patient underwent immediate open nephrectomy, stent was removed and colon was repaired. Song et al. [20] reported three types of fistula after metal stent placements, including ureteroarterial fistula, ureteroenteral fistula, and ureterovaginal fistula. When a patient with a metal stent visits the emergency room with a gross hematuria, fistula should be highly suspected.

Generally, metal stents are contraindicated in renal stone formers. We used metal stent in four ureter stone cases, but the all patients were recurred cases after ureteroplasty or endopyelotomy. According to Wang et al. [21], it is not recommended to use metal stents in patients with stones because it is more likely to form encrustation and stones. Sountoulides et al. [2] also pointed out that most encrustation was occurred in former stone disease patients.

The impact of our findings is limited by retrospective nature and relatively small sample size. However, this is not a small number in the paper about metal stent, especially when limited to benign strictures. And baseline characteristics are not comparable in radiation treatment history and stricture length between two groups. But we analyzed the risk factors with multivariate analysis and able to offset some of the limitations. Multivariate analysis provides comprehensive information between the two groups and it was verified that there was no difference in success rates between two metal stents.

The treatment outcomes from mesh and thermo-expandable stents for chronic benign ureteral strictures were comparable. Although the primary and overall success rates were not significantly different between the stent groups, the thermo-expandable stent yielded more favorable results for primary success. Non-pelvic ureteral stricture, prior radiation therapy, and previous PCN had a negative effect on success. Longer stents had a positive effect on stent success. Both stents are suitable for treating chronic benign ureteral strictures, but more systematic and detailed research is needed to estimate success rates. There is no established optimal indication or strategy for selecting metal stents, but we expect our findings to contribute to that effort.

## Conclusions

Both mesh and thermo-expandable stents are useful as a treatment for chronic benign ureteral strictures. There was no significant difference between the primary and overall success rates of the both stents, especially in the first year of stent insertion.

## Additional files

Additional files 1: **Table S1.** Risk factors affecting success rates for covered mesh stent (DOCX 19 kb)

Additional files 2: **Table S2.** Risk factors affecting success rates for thermo-expandable stent (DOCX 19 kb)

## Abbreviations

DJ: Double J stenting
PCN: Percutaneous nephrostomy
RT: Radiation therapy
UU: Ureter units
UVJ: Ureterovesical junction
