# Supplementary material for: Long-term outcomes of two types of metal stent for chronic benign ureteral strictures
Source: BMC Urol. 2019 May 6;19:34. doi: 10.1186/s12894-019-0465-5 (PMC6501332; doi:10.1186/s12894-019-0465-5)
Supplement: Supplementary file 1 — Table S1. Risk factors affecting success rates for covered mesh stent (DOCX 19 kb) [file 12894_2019_465_MOESM1_ESM.docx]

Table S1. Risk Factors Affecting Success Rates for Covered mesh stent

|  | *Primary* | | *Overall* | |
| --- | --- | --- | --- | --- |
|  | *HR* | p*-value* | *HR* | p*-value* |
| Age (yr) | 0.983 | 0.464 | 0.966 | 0.208 |
| Gender |  |  |  |  |
| Male | 1.000 | ─ | 1.000 | ─ |
| Female | 0.136 | 0.024^*^ | 0.401 | 0.367 |
| Stricture location |  |  |  |  |
| Pelvic ureter | 1.000 | ─ | 1.000 | ─ |
| Non-pelvic ureter^a^ | 3.940 | 0.116 | 7.498 | 0.117 |
| Stricture length (cm) |  |  |  |  |
| ≤10 cm | 1.000 | ─ | 1.000 | ─ |
| >10 cm | 1.146 | 0.891 | 1.475 | 0.704 |
| Stent length |  | 0.783 |  | 0.596 |
| <10 cm | 1.000 | ─ | 1.000 | ─ |
| 10–15 cm | 0.602 | 0.580 | 1.548 | 0.655 |
| >15 cm or multiple^b^ | 0.855 | 0.894 | 0.573 | 0.696 |
| Prior radiation therapy |  |  |  |  |
| No | 1.000 | ─ | 1.000 | ─ |
| Yes | 2.098 | 0.429 | 11.694 | 0.181 |
| Previous PCN |  |  |  |  |
| No | 1.000 | ─ | 1.000 | ─ |
| Yes | 3.013 | 0.103 | 2.960 | 0.202 |
| Balloon dilatation |  |  |  |  |
| No | 1.000 | ─ | 1.000 | ─ |
| Yes | 1.860 | 0.369 | 1.823 | 0.553 |

^*^Statistically significant (*p* < 0.05)

^a^upper ureteral stricture only

^b^more than two stents

HR = hazard ratio; PCN = percutaneous nephrostomy
