# Supplementary material for: Long-term outcomes of two types of metal stent for chronic benign ureteral strictures
Source: BMC Urol. 2019 May 6;19:34. doi: 10.1186/s12894-019-0465-5 (PMC6501332; doi:10.1186/s12894-019-0465-5)
Supplement: Supplementary file 2 — Table S2. Risk factors affecting success rates for thermo-expandable stent (DOCX 19 kb) [file 12894_2019_465_MOESM2_ESM.docx]

Table S2. Risk Factors Affecting Success Rates for Thermo-expandable stent

|  | *Primary* | | *Overall* | |
| --- | --- | --- | --- | --- |
|  | *HR* | p*-value* | *HR* | p*-value* |
| Age (yr) | 1.024 | 0.374 | 1.005 | 0.854 |
| Gender |  |  |  |  |
| Male | 1.000 | ─ | 1.000 | ─ |
| Female | 2.072 | 0.425 | 0.999 | 0.999 |
| Stricture location |  |  |  |  |
| Pelvic ureter | 1.000 | ─ | 1.000 | ─ |
| Non-pelvic ureter^a^ | 6.134 | 0.048^*^ | 2.662 | 0.303 |
| Stricture length (cm) |  |  |  |  |
| ≤10 cm | 1.000 | ─ | 1.000 | ─ |
| >10 cm | 6.946 | 0.177 | 0.166 | 0.241 |
| Stent length |  | 0.073 |  |  |
| <10 cm | 1.000 | ─ |  |  |
| 10–15 cm | 0.704 | 0.706 |  |  |
| >15 cm | 0.010 | 0.037^*^ |  |  |
| Prior radiation therapy |  |  |  |  |
| No | 1.000 | ─ | 1.000 | ─ |
| Yes | 11.184 | 0.069 | 1.920 | 0.651 |
| Previous PCN |  |  |  |  |
| No | 1.000 | ─ | 1.000 | ─ |
| Yes | 10.192 | 0.013^*^ | 4.390 | 0.087 |
| Balloon dilatation |  |  |  |  |
| No | 1.000 | ─ | 1.000 | ─ |
| Yes | 1.436 | 0.662 | 0.532 | 0.524 |

^*^Statistically significant (*p* < 0.05)

^a^upper ureteral stricture only

HR = hazard ratio; PCN = percutaneous nephrostomy
